# Porous Single-Crystalline Rare Earth Phosphates Monolith to Enhance Catalytic Activity and Durability

**DOI:** 10.3390/molecules30020331

**Published:** 2025-01-15

**Authors:** Wenting Li, Lingting Ye, Chaoyang Tu, Kui Xie

**Affiliations:** 1 Key Laboratory of Design and Assembly of Functional Nanostructures, Fujian Institute of Research on the Structure of Matter, Chinese Academy of Sciences, Fuzhou 350002, China; 2 University of Chinese Academy of Sciences, Beijing 100039, China; 3 Fujian College, University of Chinese Academy of Sciences, Fuzhou 350002, China; 4 School of Mechanical Engineering, Shanghai Jiao Tong University, 800 Dongchuan Road, Shanghai 200240, China

**Keywords:** porous single-crystalline, rare earth phosphate, oxygen defect, activity, stability

## Abstract

Rare earth phosphate (XPO_4_) is an extremely important rare earth compound. It can exhibit excellent activity and stability in catalytic applications by modifying its inherent properties. Porous single-crystalline (PSC) PrPO_4_ and SmPO_4_ with a large surface area consist of ordered lattices and disordered interconnected pores, resulting in activity similar to nanocrystals and stability resembling bulk crystals. Herein, we present a study in which centimeter-scale PSC PrPO_4_ and SmPO_4_ monoliths were developed and oxygen defects in the crystal lattice were stabilized using single-crystal nature to synergistically improve catalytic activity in the oxidative dehydrogenation of ethane (ODE). The surface structure of the oxygen vacancies with unsaturated coordination is favorable for the adsorption and activation of ethane. The PSC PrPO_4_ and SmPO_4_ monoliths showed favorable performance with ~51% conversion of C_2_H_6_ and ~19% yield of C_2_H_4_ at 600 °C, while also exhibiting superior long-term stability during the catalytic process over a period of 115 h. In the presented work, we investigate a practical method for development and application in single-crystalline porous rare earth phosphate materials.

## 1. Introduction

Rare earth phosphate (XPO_4_) combines phosphate lattices with rare earth ions. It has applications in optics, catalysis, and electrochemistry [1]. XPO_4_ has excellent thermal properties and chemical stability and can thus be used stably in high-temperature environments [2,3]. Its unique lanthanide electron configuration interacts with the anionic coordination polyhedral to accommodate the active site and maintain an excellent internal structure at high temperatures. Therefore, its inherent properties can be changed by synergistically controlling its microstructure and defects to exhibit high activity and stability in catalyst applications.

Porous single-crystalline (PSC) materials combine the characteristics of single crystals with interconnected pore structures, creating structures with well-defined surfaces that are dynamically trapped. Notably, PSC materials are not single-crystal materials but rather a type of porous material with characteristics similar to single crystals. In order to highlight the structural characteristics of porous and single-crystal materials, we refer to them as porous single-crystalline materials. A porous structure can provide a larger surface area sufficient for chemical reactions [4]. A surface with well-defined structures can afford advantages to the construction and recognition of active sites that can adjust the chemical interaction with the adsorbed substance by altering the electronic structure at the surface. Moreover, at the macroscale, the porous structure promotes phase diffusion and enhances the chemical interaction between reacting species and active sites in the porous structure. PSC XPO_4_ is able to maintain intrinsic thermal stability and provide clear surface construction to form active sites, facilitating the formation of oxygen defects.

The price of crude oil and the practicability of ethane in shell gas have increased interest in replaceable processes for the production of ethylene [5]. The oxidative dehydrogenation of ethane (ODE) may be one potential method for replacing traditional steam cracking. It has lower cost, increased safety, and lower impact on the environment. The ODE also overcomes the disadvantages of ethane dehydrogenation without oxygen. The reaction process changes to the exothermic reaction by introducing an oxidant, which reduces the temperature of the reaction, improves the conversion of alkanes, and avoids carbon deposition. The ODE process requires less equipment, which can greatly reduce investment costs [6,7]. However, the ODE has yet to reach an industrialized application due to its low reaction selectivity; the main reason for this limitation is the fact that it is easy for the oxidizer in the reaction atmosphere to further oxidize ethylene to oxycarbide (CO_x_) and H2O [8]. Therefore, this issue presents a significant challenge in exploring ODE catalysts with high activity, high yield, and long lifespan.

Here, we fabricated centimeter-scale PSC PrPO_4_ and PSC SmPO_4_ monoliths that combine an ordered lattice with disordered interconnected pores through synergistic effects to form consecutive surfaces that are dynamically trapped in the high energy state. Thus, these single-crystal structures have inherent stability akin to that of bulk single-crystalline materials; however, they also have activity identical to that of nanocrystals. The single-crystalline properties contribute to the generation and stabilization of oxygen defects, further improving catalytic activity. We proposed the good performance of oxidative dehydrogenation of ethane and stable operation at 600 °C over 115 h. This work highlights a new application of rare earth phosphates.

## 2. Results and Discussion

**Figure 1:**
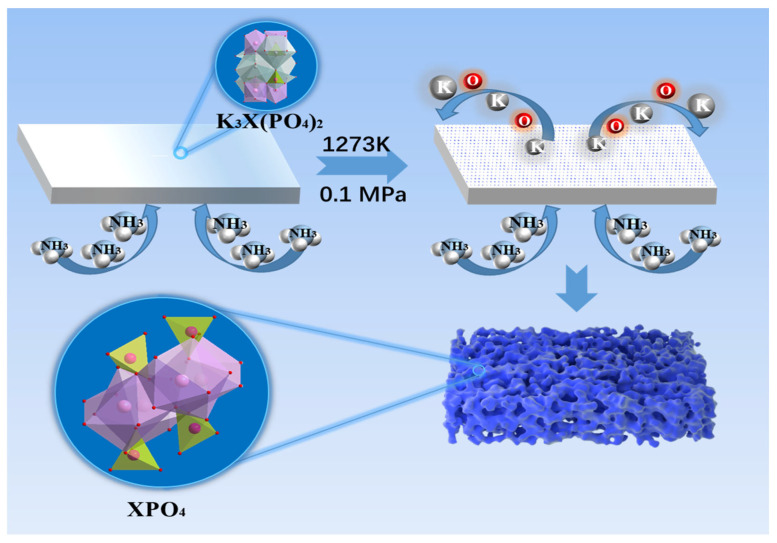
Schematic diagram depicting the formation of the porous XPO_4_ (X = Pr, Sm) single-crystal monolith.

Figure 1 shows a schematic illustration of PSC XPO_4_ (X = Pr, Sm) growth using the lattice reconstruction strategy. We began the experiment by growing K_3_Pr(PO_4_)_2_ and K_3_Sm(PO_4_)_2_ single crystals and then treated K_3_Pr(PO_4_)_2_ and K_3_Sm(PO_4_)_2_ (with facets (001)) in NH_3_ atmosphere at 1000–1100 °C to develop PSC PrPO_4_ and PSC SmPO_4_ monoliths with the facets of (111) using a lattice reconstruction strategy at a high temperature (Figure S1, Supporting Information) [9]. The K-O bond fracture, K element, and partial O element removal from the lattice lead to the collapse of the lattice, and with lattice reconstruction, a porous single crystal is ultimately formed. In the system, it is the operation temperature that mainly drives the removal of target elements (K and partial O) from the mother phase, which facilitates solid–solid transformation for growing PSC PrPO_4_ and SmPO_4_ monoliths [10,11]. This method not only applies to obtain PSC PrPO_4_ and SmPO_4_ but also generates other porous rare earth phosphate single crystals by changing the temperature and pressure.

**Figure 2:**
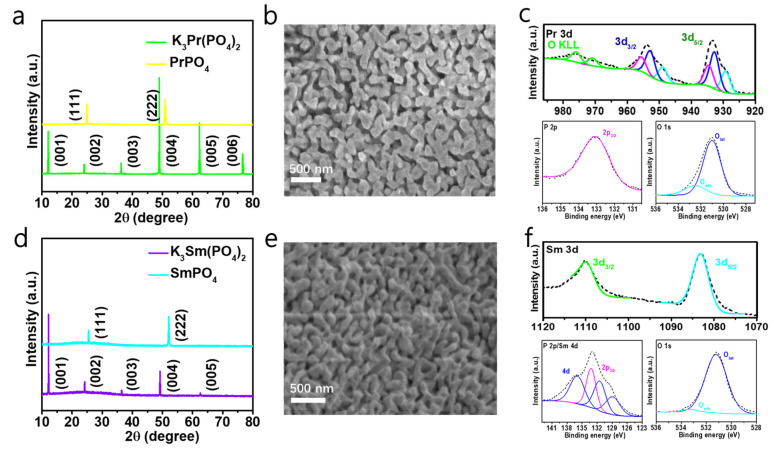
X-ray diffraction (XRD) pattern with (001) facets of K_3_Pr(PO_4_)_2_ (**a**) and K_3_Sm(PO_4_)_2_ (**d**) and (111) facets of PrPO_4_ (**a**) and SmPO_4_ (**d**). Scanning electron microscope (SEM) image of PSC PrPO_4_ (**b**) and SmPO_4_ (**e**) with (111) facets. X-ray photoelectron spectroscopy (XPS) of PSC PrPO_4_ (**c**) and SmPO_4_ (**f**) with (111) facets.

Figure 2a,d show the XRD patterns of the PSC PrPO_4_ and PSC SmPO_4_ (both with facets (111)) monoliths, which demonstrate the single-crystalline features. The peaks of the spectra are sharp, which indicates the good crystallinity of PSC PrPO_4_ and SmPO_4_. The porosity was tested to be ~87.50% and ~87.15% in the PSC PrPO_4_ and SmPO_4_ monoliths. K_3_Pr(PO_4_)_2_ and K_3_Sm(PO_4_)_2_ single crystals both have a space group of P 1 21/m 1 (11)—monoclinic with a = 7.458(1) Å, b = 5.632(1) Å, c = 9.551(1) Å, β = 90.87(1)°, and a = 7.4347(5) Å, b = 5.6270(5) Å, c = 9.4919(5) Å, β = 90.870(6)°, respectively [12,13]. Figure S2 shows the energy-dispersive spectroscopy (EDS) results for the K_3_Pr(PO_4_)_2_ and K_3_Sm(PO_4_)_2_ single crystals, which match well with PSC PrPO_4_ and SmPO_4_ shown in Figure S4, except for the K element. The corresponding mapping images indicate that the elements are evenly distributed in the skeleton. The PSC PrPO_4_ and SmPO_4_ monoliths both have the same space group of P 1 21/n 1 (14)—monoclinic with a = 6.7596(8) Å, b = 6.9812(10) Å, c = 6.4344(9) Å, β = 103.53(1)°, and a = 6.6818(12) Å, b = 6.8877(9) Å, c = 6.3653(9) Å, β = 103.86(1)°, respectively [14]. Figure 2b,e, and Figure S5 show the uniform microstructure of the porous PrPO_4_ and SmPO_4_ single crystals with a mean pore size of roughly 130 nm and suggest excellent three-dimensional connectivity in the porous single-crystalline material. We used the mercury injection method to analyze the porosity levels of commercial PrPO_4_, commercial SmPO_4_, PSC PrPO_4_, and PSC SmPO_4_, and the Supplementary Data results are shown in Table S1. It can be seen that the porosities of PSC PrPO_4_ and PSC SmPO_4_ are both higher than that of the commercial samples, and the porosity of the two porous single crystals is similar. Porous single crystals can provide larger surface areas sufficient for chemical reactions.

Figure 2c shows that the spin–orbit splitting (SOS) in PSC PrPO_4_ leads to three spin–orbit double peaks, in which the binding energies are approximately 929.4 eV/949.0 eV, 932.8 eV/953.0 eV, and 934.6 eV/955.7 eV, respectively. These double peaks represent the Pr 3d_5/2_ and Pr 3d_3/2_ components of the spectrum [15]. The characteristic oxygen Auger peaks (976.2 eV and 971.0 eV) are labeled OKLL in the spectrogram [16,17]. The lattice oxygen (O_lat_) and adsorbed oxygen (O_ad_) of PrPO_4_ contribute to the XPS peak of O 1s at 531.0 eV and 532.5 eV, respectively [18,19]. The XPS peak of lattice oxygen appears after 530.5 eV due to the increase in binding energy caused by the bonding of oxygen atoms not only with rare earth but also with phosphorus atoms in rare earth phosphate. The centered peak of P on 133.1 eV corresponding to the electronic orbit of P 2p_3/2_ is attributed to P^5+^ in PrPO_4_ [18]. Figure 2f shows that the SOS is present in PSC SmPO_4_. The Sm 3d possesses two spin–orbit peaks, one at 1110.1 eV, corresponding to 3d_3/2_, and one at 1083.1 eV, corresponding to 3d_5/2_ [20]. PSC SmPO_4_ also has two XPS peaks of oxygen at 531.2 eV and 533.3 eV. The binding energy of the Sm 4d and P 2p orbitals in SmPO_4_ coincides, with the XPS peaks in SmPO_4_ of P 2p at approximately 133.2 eV corresponding to 2p_3/2_ and Sm 4d at approximately 129.0 eV/131.6 eV/136.0 eV [18,21]. It is worth mentioning that PSC PrPO_4_ and SmPO_4_ contain the same types of elemental electron orbitals as the parent single crystals shown in Figure S3, which suggests that despite lattice reconstruction causing lattice collapse, the chemical environment in which the atoms are present may not have changed to a considerable extent.

**Figure 3:**
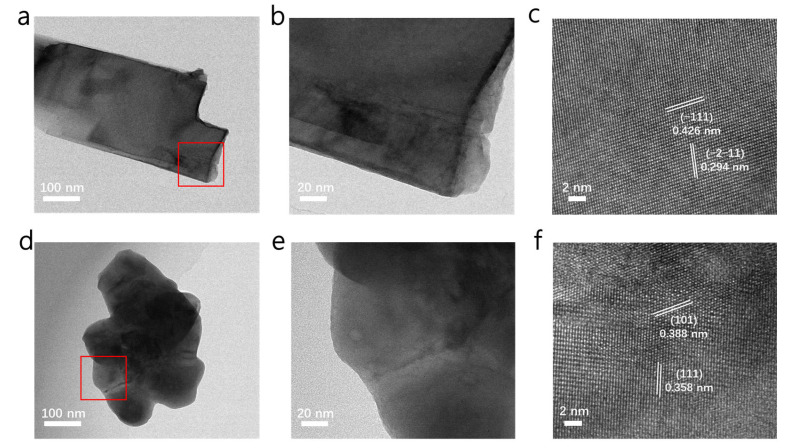
TEM images of PSC PrPO_4_ (**a**) and PSC SmPO_4_ (**d**); enlarged TEM images of the edges of PSC PrPO_4_ (**b**) and PSC SmPO_4_ (**e**); HRTEM images of PSC PrPO_4_ (**c**) and PSC SmPO_4_ (**f**).

We ground the PSC PrPO_4_ and SmPO_4_ monoliths into a powder, dispersed them with ethanol, dripped them on copper wire mesh, and used transmission electron microscopy (TEM) to obtain in-depth information about them. Figure 3a–c show the microstructure of PSC PrPO_4_, which also demonstrates the single-crystalline characteristics in the porous structures. The d-spacing of 0.294 nm and 0.426 nm match well with (−2–11) and (−111) facets shown in Figure 3c and Figure S6a, respectively. Moreover, we used EDS to characterize the elemental information of the corresponding position in the TEM samples shown in Figure S6b. The peak position and peak intensity of the elements are essentially consistent with those of the centimeter-size porous single crystals shown in Figure S4a. Figure 3d–f show the microstructure of PSC SmPO_4_. Its shape is different compared to PSC PrPO_4_ due to the fact that the sample was prepared by hand and ground into powder; however, this difference in shape did not affect its single-crystal characteristics. The d-spacing of 0.358 nm and 0.388 nm match well with the (111) and (101) facets shown in Figure 3f and Figure S6c, respectively. The EDS analysis of the corresponding position in the TEM samples in Figure S6d show that the peaks of the elements are both essentially consistent with the centimeter-sizeed porous single crystals shown in Figure S4c, which indicates that PSC PrPO_4_ and SmPO_4_ can both maintain a good single-crystal state with the presence of the pore structure.

**Figure 4:**
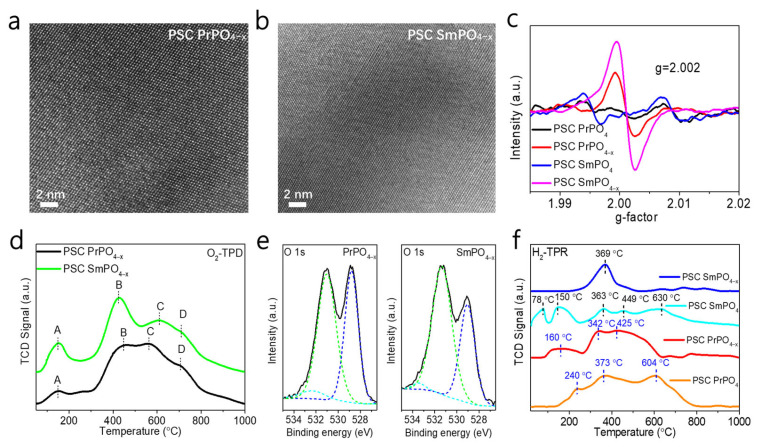
HRTEM images of PSC PrPO_4−x_ (**a**) and PSC SmPO_4−x_ (**b**); EPR spectra image (**c**) of unrestored and reduced samples; O_2_-TPD (**d**) with four O-type desorption peaks (labeled with A, B, C, and D) and O 1 s XPS images (**e**) of PSC PrPO_4−x_ and SmPO_4−x_; and H_2_-TPR (**f**) of PSC PrPO_4_, PrPO_4−x_, SmPO_4_, and SmPO_4−x_.

PSC PrPO_4_ and SmPO_4_ with oxygen defects were obtained through the reduction in hydrogen (H_2_) atmosphere at 500 °C for 2 h. Figure 4a,b show the HRTEM images of the reduced PSC PrPO_4_ and SmPO_4_ (PrPO_4−x_ and SmPO_4−x_). The surfaces of the samples are pure, and the atomic composition is very clear, which is conducive to providing active sites for catalytic reactions. In addition, it was found that the reduced PSC SmPO_4_ is more tolerant to high-energy electron beams and has a clearer and more stable lattice structure than the non-reduced sample. The samples in the TEM test were not prepared directionally, and the crystal lattice orientation of the reduced samples differed from that of the unreduced samples. The d-spacing of 0.326 nm/0.344 nm match well with the (200) facet/(020) facet in PSC PrPO_4−x_ and that of 0.267 nm/0.344 nm match well with the (121) facet/(020) facet in PSC SmPO_4−x_ shown in Figures S7b,e, respectively. The single-crystal properties were well preserved before and after reduction, thus exhibiting the features of a porous single-crystalline material. The EDS analysis of the corresponding position matches well with the centimeter-sized, porous single crystals in Figure S7c,f. Figure 4c shows the EPR spectra in the signals with g values of 2.002 for PSC PrPO_4−x_ and SmPO_4−x_, which can be ascribed to oxygen defects [22]. In contrast, there are almost no oxygen defect signals on PSC PrPO_4_ and SmPO_4_. These results show that there are numerous oxygen defects in PSC PrPO_4−x_ and SmPO_4−x_. Figure 4d represents the O_2_-TPD on the PSC PrPO_4−x_ and SmPO_4−x_ from 50 °C to 1000 °C. Four O-type desorption peaks (labeled with A, B, C, and D) both appeared in two samples with the increase in temperature. The A peak can be ascribed to the desorption of the oxygen physically adsorbed on a surface below 200 °C. The B peak may be due to the desorption of chemisorbed substances, which may be related to surface oxygen defects. The high-temperature C and D peaks (higher than 550 °C) account for the desorption of the surface lattice oxygen [23,24]. These results further indicate the presence of oxygen defects on PSC PrPO_4−x_ and SmPO_4−x_.

Figure 4e provides the O 1s XPS spectra of PSC PrPO_4−x_ and SmPO_4−x_, which can be divided into three peaks at the binding energies of ~528.8 eV (O_lat_), 531.0 eV, and 532.4 eV (O_ad_) in PSC PrPO_4−x_ and ~529.1 eV (O_lat_), 531.3 eV, and 533.5 eV (O_ad_) in PSC SmPO_4−x_. It can be found that the O 1 s XPS peak of PSC PrPO_4−x_ and SmPO_4−x_ was shifted compared with the unreduced samples. A new oxygen species was produced that corresponds to the nucleophilic oxygen species compared with the XPS of PSC PrPO_4_ and PSC SmPO_4_ in Figure 2c,f, which are generally considered to be the active oxygen species generated during the selective oxidation of alkanes [25,26,27]. The lattice oxygen species (~529 eV) in PSC PrPO_4−x_ and SmPO_4−x_ is the electrophilic oxygen species. The relative content of electrophilic oxygen species in PSC PrPO_4−x_ and PSC SmPO_4−x_ is lower than that of the unreduced samples, which is also conducive to the catalytic activity of the ODE reaction. Figure S8 shows the XPS spectra of the other two elements in PSC PrPO_4−x_ and PSC SmPO_4−x_. The XPS spectra of metal elements did not change significantly before and after reduction; however, a new peak also appeared in the XPS spectra of P 2p, indicating that oxygen defects may be primarily located in P-O bonds. As shown in Figure 4f, we proceeded with Temperature Programmed Reduction (TPR) of PSC PrPO_4_ and PSC SmPO_4_ under an H_2_ atmosphere (10% H_2_ and 90% Ar). Results indicate that the oxygen defects in the lattice usually appear under this atmosphere from 70 °C to 630 °C. The increase in hydrogen concentration under this atmosphere can effectively reduce the temperature of oxygen defect formation in PSC PrPO_4_ and SmPO_4_. Moreover, the reduction peak position of PSC PrPO_4−x_ and PSC SmPO_4−x_ significantly shifted to the direction of low temperature (from 604 °C to 425 °C and from 630 °C to 369 °C, respectively), indicating that hydrogen reduction treatment can significantly improve the reduction performance of PSC PrPO_4_ and PSC SmPO_4_ while also improving lattice oxygen activity. The PSC PrPO_4_ and SmPO_4_ monoliths exhibited higher lattice oxygen activity in contrast with PrPO_4_ and SmPO_4_ purchased from a pharmaceutical company, as shown in Figure S9, within the temperature range of the TPR profile. We took the ODE reaction as an example for exploring the impact of PSC PrPO_4−x_ and PSC SmPO_4−x_ on the activity and durability of catalysis.

**Figure 5:**
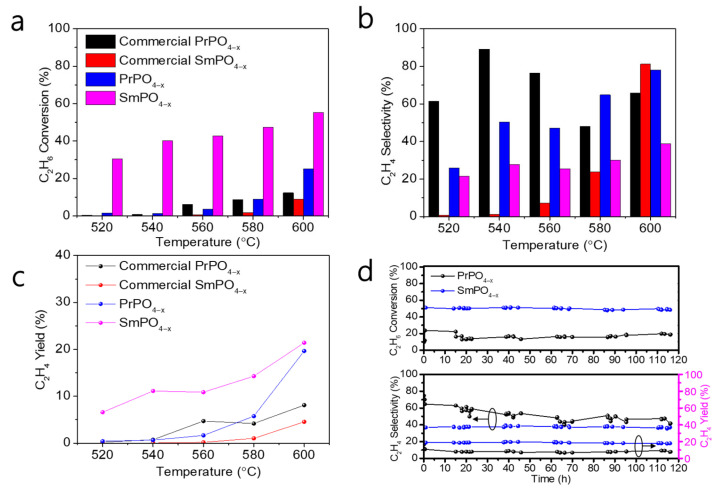
The conversion of ethane (C_2_H_6_) (**a**); the selectivity (**b**) and yield (**c**) of ethylene (C_2_H_4_) in the commercial PrPO_4−x_, commercial SmPO_4−x_, PSC PrPO_4−x_, and PSC SmPO_4−x_ from 520 °C to 600 °C (10% C_2_H_6_, 10% O_2_, argon as balanced gas; pressure at 0.1 MPa, space velocity with 18,000 mL g^−1^ h^−1^; each point is a single measurement); (**d**) stability in PSC PrPO_4−x_ and PSC SmPO_4−x_ at 600 °C with the conversion of C_2_H_6_ and the selectivity and yield of C_2_H_4_ (10% C_2_H_6_, 10% O_2_, argon as balanced gas; pressure at 0.1 MPa; space velocity with 18,000 mL g^−1^ h^−1^).

We purchased commercial PrPO_4_ and SmPO_4_ and performed the same restoration process before the reaction. Figure 5a–c show the conversion of ethane and the selectivity and yield of ethylene in the ODE process of commercial PrPO_4−x_, commercial SmPO_4−x_, PSC PrPO_4−x_, and PSC SmPO_4−x_ from 520 °C to 600 °C. The ethane conversion of these samples gradually increased with temperature, and the increasing ethane conversion trend in the PSC samples was higher than that of the commercial samples. The ethane conversion of PSC PrPO_4−x_ and PSC SmPO_4−x_ ultimately reached roughly 25% and 55% at 600 °C, respectively, which is an around two- and seven-fold improvement compared to that of commercial PrPO_4−x_ and commercial SmPO_4−x_. PSC PrPO_4_ and PSC SmPO_4_ are more likely to generate more active sites than the corresponding commercial materials, which is conducive to the adsorption and activation of ethane. The ethylene selectivity of PSC PrPO_4−x_ and PSC SmPO_4−x_ is not as high as their ethane conversion. This result may be due to the fact that although the porous single-crystal surface produces abundant oxygen defects, which are conducive to adsorption and deionization in gas molecules, active oxygen atoms also cause extensive oxidation, which leads to a reduction in ethylene selectivity. The ethylene yield of PSC PrPO_4−x_ and SmPO_4−x_ increased to 19% and 21% at 600 °C, which is roughly a 2.4- and 5.5-fold improvement compared to that of commercial PrPO_4−x_ and SmPO_4−x_. Although active oxygen atoms in the samples can cause extensive oxidation that in turn reduces the selectivity of ethylene, the clear active surface structure of the porous single-crystalline materials is conducive to regulating the interaction between adsorbed species and active sites, ultimately significantly improving ethylene yield [28]. Porous single-crystalline materials with oxygen defects effectively improve ethane conversion and maintain the good selectivity of the product.

We continued the oxidative ethane dehydrogenation reaction with PSC PrPO_4−x_ and SmPO_4−x_ at 600 °C. The conversion of ethane was low and the selectivity of ethylene was high in the initial PSC PrPO_4−x_ reaction, as shown in Figure 5d. As the reaction progresses, the conversion of ethane increases whereas the selectivity of ethylene decreases. The changing trend of the two gradually reached stability. The conversion of ethane and the selectivity of ethylene in PSC SmPO_4−x_ remained constant. The yield of PSC PrPO_4−x_ and PSC SmPO_4−x_ remained stable in the long-term reaction. This phenomenon may be due to the electron transfer of the element Pr on PSC PrPO_4−x_. Pr is a rare earth element that is prone to electron transfer in an identical manner to Ce. In the initial stages of the reaction, oxygen vacancies serve as active sites for selective oxidation, and as the reaction progresses, electron transfer also occurs on Pr to facilitate the reaction. However, the active sites on Pr may be non-selective active sites; therefore, although C_2_H_6_ conversion increases, C_2_H_4_ selectivity decreases. Overall, Figure 5d shows the conversion of ethane, and the selectivity and yield of ethylene are both stable even after a period of 115 h, which indicates that the two porous single crystals show potential in the ODE reaction. Figure S10 shows the Raman spectrum from 1000 to 2000 cm^−1^ tests on PSC PrPO_4−x_ and SmPO_4−x_ after the reaction, which demonstrates that the carbon deposits are almost non-existent. The porous single crystal may work effectively in resisting coking and sintering of the surfaces at higher temperatures. The TEM images of PSC PrPO_4−x_ and SmPO_4−x_ following the long-term reaction, demonstrated in Figure S11, show a clear lattice and they maintain good single-crystalline properties. Figure S12 shows the XPS spectrum of PSC PrPO_4−x_ and SmPO_4−x_ following the long-term reaction, with no new spin–orbit peaks compared with the reduced samples and an increase in the relative content of nucleophilic oxygen (~531 eV). The molecular structure and single-crystalline properties of PSC PrPO_4−x_ and SmPO_4−x_ remained stable, and the unsaturated coordination structure provided by oxygen defects as the active site facilitated activation for the ODE reaction. The stability and test duration of reduced PSC PrPO_4_ and SmPO_4_ are good, which may be attributed to the stable structure, which allows oxygen defects to remain stable at high temperatures for long periods, and the clear surface structure regulates the interaction between the active site and adsorbed species.

**Figure 6:**
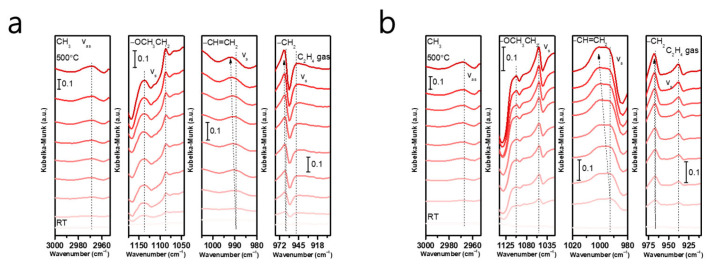
In situ infrared absorption (IR) spectrometry of ethane and oxygen activation on the surface of PSC PrPO_4−x_ (**a**) and PSC SmPO_4−x_ (**b**) from room temperature (RT) to 500 °C.

We utilized in situ IR spectroscopy to examine the ethane activation process on the surface of the PSC PrPO_4−x_ and PSC SmPO_4−x_ monoliths, as shown in Figure 6, from room temperature to 500 °C. The C−H bond showed a gradually visible and clear asymmetric stretching vibration in ethane (both at 2967.8 cm^−1^) with the increase in temperature, which indicates that the C−H bond is effectively activated and alters to a higher-energy state [29,30,31,32]. Moreover, the vibration peaks at 1087.6 cm^−1^/1052.9 cm^−1^ are ascribed to the C−O bond; in addition, 1137.8 cm^−1^/1103.1 cm^−1^ is ascribed to the C−C bond in ethoxy (−OCH_2_CH_3_) species, which are considered to be pivotal intermediates in the ODE catalytic reaction [33,34,35]. The stretching vibration intensity of the two bonds gradually increases with the increase in temperature. Furthermore, the C=C bond and C−H bond in ethylene with stretching vibration are clearly blue-shifted, which indicates that these two bonds are effectively activated to higher-energy states. The infrared peak indicates that the amount of ethylene gas is also increasing [29,35,36,37,38,39].

From the in situ IR spectroscopy results, we hypothesized the feasible path of the catalysts for the ODE reaction. The C−H bond of ethane adsorbed on the surface of PSC PrPO_4−x_ and PSC SmPO_4−x_ catalysts will first be activated and the lattice oxygen on the surface is involved to form adsorbed ethoxy species. After the removal of β-H from the ethoxy, ethylene is produced. This process is consistent with the Mars–van Krevelen (MvK) mechanism, whereby the surface-active oxygen activates the first C−H bond in the alkane, which is the rate-determining step in the ODE reaction [40].

## 3. Experiment

### 3.1. Material Preparation

Using the molten salt method, we grew the mother phase of K_3_Pr(PO_4_)_2_ and K_3_Sm(PO_4_)_2_ single crystals as substrates to transform them into porous single-crystalline PrPO_4_ and SmPO_4_ monoliths in a horizontal alumina chamber equipped with mass flow meters in an NH_3_ atmosphere (300 mL/min, 100% NH_3_) at 1000–1100 °C [41]. We then reduced the samples to prepare PSC PrPO_4−x_ and PSC SmPO_4−x_ by flowing H_2_ gas (20 mL/min, 100% H_2_) in a horizontal alumina chamber equipped with mass flow meters to maintain the system’s temperature at 500 °C for roughly 2 h.

### 3.2. Material Characterization

We used X-ray diffraction (XRD) to examine the phase formation (Cu-Ka, Mniflex 600, Rigaku, Japan) and X-ray photoelectron spectroscopy (XPS) to analyze the chemical state of the elements in the samples (ESCALAB 250Xi, Thermo Fisher, Waltham, MA, USA). A field-emission scanning electron microscope (SU-8010, FE-SEM, Hitachi, Tokyo, Japan) and field-emission transmission electron microscope (Tecnai G2 F20 S-TWIN TMP, FE-TEM, FEI, Hillsboro, OR, USA) were used to analyze the morphologies of the samples. The oxygen defects in the samples were analyzed using an electron paramagnetic resonance (EPR) spectrometer (EPR200M, CIQTEK, Hefei, China). The Raman spectra were obtained with a laser confocal Raman spectrometer (Lambda950, PerkinElmer, Waltham, MA, USA). The chemisorbed instruments (AMI-300, Altamira Instruments, Cumming, GA, USA) were used to test the H_2_-TPR and O_2_-TPD. IR spectrometer (VERTEX 70, Bruker, Billerica, MA, USA) was used to perform the Diffuse Reflectance Infrared Fourier Transform Spectroscopy (DRIFTS) measurements. A Mercury injection instrument (AutoPore IV 9500, Micromeritics, Norcross, GA, USA) was used for porosity analysis.

### 3.3. Catalytic Test

We performed the catalytic tests under atmospheric pressure (about 0.1 MPa) in a tubular reaction system that includes a flow quartz micro-reactor with an inner diameter of 5 mm. The porous single-crystalline material was preheated at the temperature of (500 °C) for roughly 0.5 h in Ar flow. In the oxidative dehydrogenation of ethane, the gas reactant consists of 10 vol% ethane (C_2_H_6_), 10 vol% oxygen (O_2_), and argon as a balance gas. The space velocity of the gas reactant is 18,000 mL g^−1^ h^−1^. The products of the reaction were analyzed using an online gas chromatograph (GC), which was configured with FID and TCD detectors (GC-2014, Shimadzu, Japan), and a 30 m packed column of CP-poraplot Q. The conversion of ethane ($X_{C_{2}H_{6}}(\%)$) and the selectivity of ethylene ($S_{C_{2}H_{4}}(\%)$) were computed using the following formulae [42]:

(1) $$X_{C_{2}H_{6}}\left(\%\right)=\frac{{\left[C_{2}H_{6}\right]}_{in}-{\left[C_{2}H_{6}\right]}_{out}}{{\left[C_{2}H_{6}\right]}_{in}}\times 100\%$$

(2) $$S_{C_{2}H_{4}}\left(\%\right)=\frac{{\left[C_{2}H_{4}\right]}_{out}}{{\left[C_{2}H_{6}\right]}_{in}-{\left[C_{2}H_{6}\right]}_{out}}\times 100\%$$

where ${\left[C_{2}H_{6}\right]}_{in}$ is the $\left[C_{2}H_{6}\right]$ molar concentration in the feed gas and ${\left[C_{2}H_{6}\right]}_{out}$ and ${\left[C_{2}H_{4}\right]}_{out}$ are the $C_{2}H_{6}$ and $C_{2}H_{4}$ molar concentrations in the products, respectively.

The ethylene field ($Y_{C_{2}H_{4}}(\%)$) was calculated as follows [43]:

(3) $$Y_{C_{2}H_{4}}(\%)=\frac{{\left[C_{2}H_{6}\right]}_{in}-{\left[C_{2}H_{6}\right]}_{out}}{{\left[C_{2}H_{6}\right]}_{in}}\times \frac{{\left[C_{2}H_{4}\right]}_{out}}{{\left[C_{2}H_{6}\right]}_{in}-{\left[C_{2}H_{6}\right]}_{out}}\times 100\%$$

## 4. Conclusions

In summary, we developed PSC PrPO_4_ and SmPO_4_ monoliths at a centimeter scale using a lattice reconstruction strategy and further constructed well-defined oxygen-deficient surface structures to enhance the catalytic activity of the ODE reaction. Oxygen-deficient surface structures with unsaturated coordination are favorable for ethane adsorption and activation. They demonstrate good performance in ethane conversion, ~51%, and ethylene yield, ~19%, without exhibiting apparent performance degradation, even after a reaction of over 115 h in duration at 600 °C. We have developed a new method to synthesize intrinsically active PSC PrPO_4_ and SmPO_4_ monoliths that can synchronously achieve stability and overall practical performance for the oxidative dehydrogenation of the ethane reaction. Our method provides a practical method for the development and application of porous rare earth phosphate single-crystalline materials.

## Data Availability

Data are contained within the article and Supplementary Materials.

## Supplementary Materials

The following supporting information can be downloaded at: https://www.mdpi.com/article/10.3390/molecules30020331/s1. Figure S1. shows the growth mechanism of PSC PrPO4 and SmPO4. Figures S2 and S4. shows the element distribution of the surfaces in K3Pr(PO4)2, K3Sm(PO4)2 single crystal monoliths, PSC PrPO4 and SmPO4. Figure S3. shows the chemical state of the surfaces in K3Pr(PO4)2 and K3Sm(PO4)2 single crystal monoliths. Figures S5 and S6 show the microstructure and element distribution of the PSC PrPO4 and SmPO4 monoliths. Figure S7 shows the microstructure and element distribution of the PSC PrPO4−x and SmPO4−x monoliths. Figure S8 shows the chemical state of the surfaces in PSC PrPO4−x and SmPO4−x. Figure S9 shows the reduction characterization of commercial PrPO4, SmPO4, PrPO4−x and SmPO4−x. Figures S10, S11 and S12 show the characterization, microstructure and chemical state of PSC PrPO4−x and PSC SmPO4−x after reaction. Table S1 shows the porosity level analysis from Mercury intrusion method of commercial PrPO4, commercial SmPO4, PSC PrPO4 and PSC SmPO4.
