# Supplementary material for: Porous Single-Crystalline Rare Earth Phosphates Monolith to Enhance Catalytic Activity and Durability
Source: Molecules. 2025 Jan 15;30(2):331. doi: 10.3390/molecules30020331 (PMC11767269; doi:10.3390/molecules30020331)
Supplement: Supplementary file 1 [file molecules-30-00331-s001.zip › molecules-3383102-supplementary.pdf]

Porous Single-crystalline Rare Earth Phosphates Monolith to Enhance Catalytic Activity and Durability

Wenting Li [1,2,3], Lingting Ye [1,2,3,*], Chaoyang Tu [1,2,3,*] and Kui Xie [1,2,3,4]

[1] Key Laboratory of Design and Assembly of Functional Nanostructures, Fujian Institute of Research on the Structure of Matter, Chinese Academy of Sciences, Fuzhou 350002, China; liwenting@fjirsm.ac.cn (W.L.); tcy@fjirsm.ac.cn (C.T.); kxie@fjirsm.ac.cn (K.X.)

[2] University of Chinese Academy of Sciences, Beijing 100039, China

[3] Fujian College, University of Chinese Academy of Sciences, Fuzhou 350002, China

[4] School of Mechanical Engineering, Shanghai Jiao Tong University, 800 Dongchuan Road, Shanghai 200240, China

* Correspondence: ltye@fjirsm.ac.cn, tcy@fjirsm.ac.cn

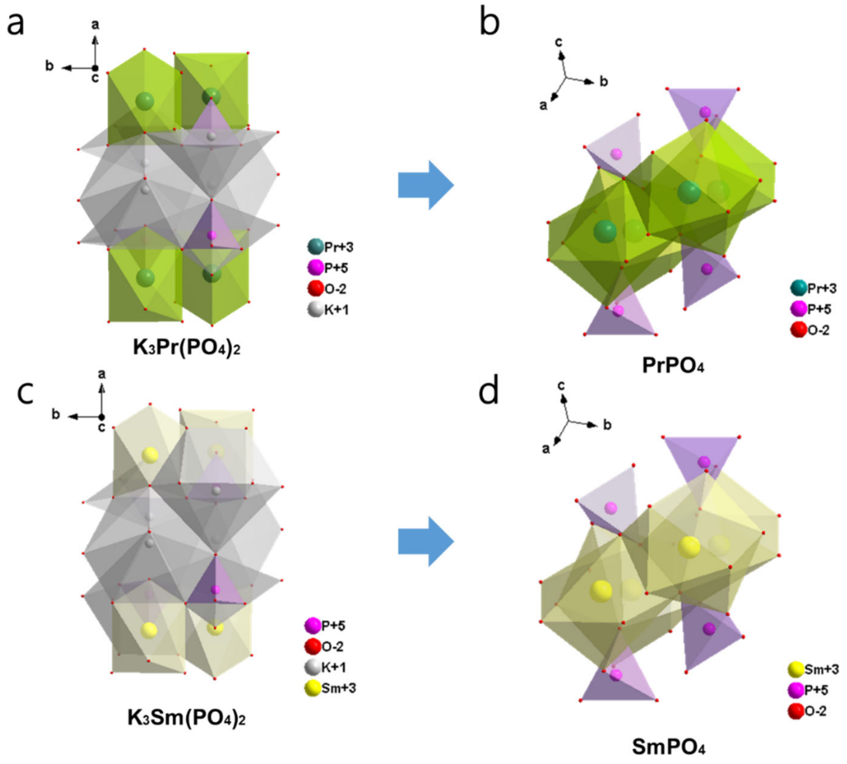

Supplementary Figure S1. Growth of porous single crystals. a–b, The growth mechanism of (001) $K_3Pr(PO_4)_2$ (a) to (111) $PrPO_4$ (b). c–d, The growth mechanism of (001) $K_3Sm(PO_4)_2$ (c) to (111) $SmPO_4$ (d).

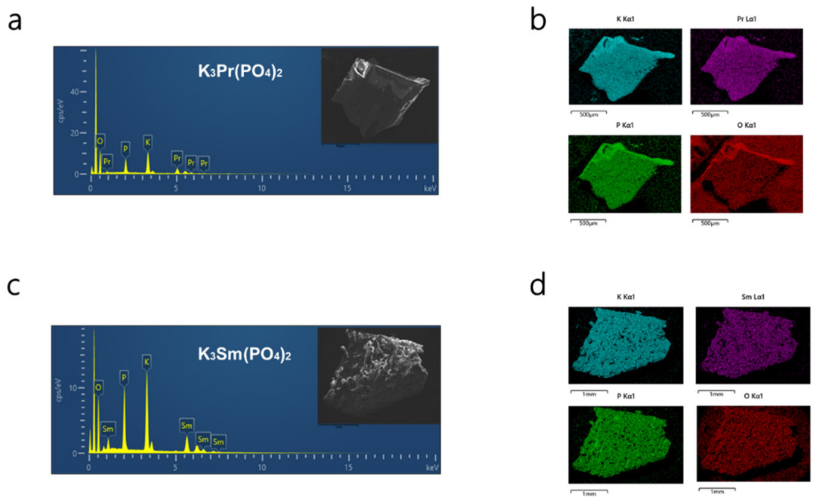

Supplementary Figure S2. Element distribution of the surfaces in $K_3Pr(PO_4)_2$ and $K_3Sm(PO_4)_2$ single crystal monoliths. a−b, The energy dispersive spectroscopy (EDS) of $K_3Pr(PO_4)_2$ and the corresponding mapping images: K Kα1, Pr Lα1, P Kα1 and O Kα1. c−d, The EDS of $K_3Sm(PO_4)_2$ and the corresponding mapping images: K Kα1, Sm Lα1, P Kα1 and O Kα1.

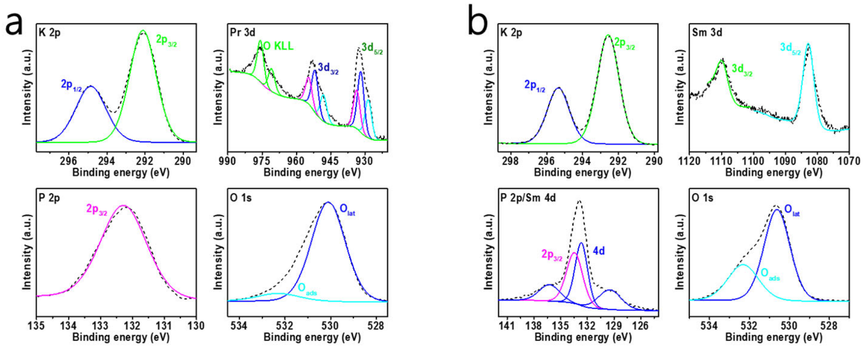

Supplementary Figure S3. Chemical state of the surfaces in $K_3Pr(PO_4)_2$ and $K_3Sm(PO_4)_2$ single crystal monoliths. a, The XPS of $K_3Pr(PO_4)_2$ single crystal. b, The XPS of $K_3Sm(PO_4)_2$ single crystal.

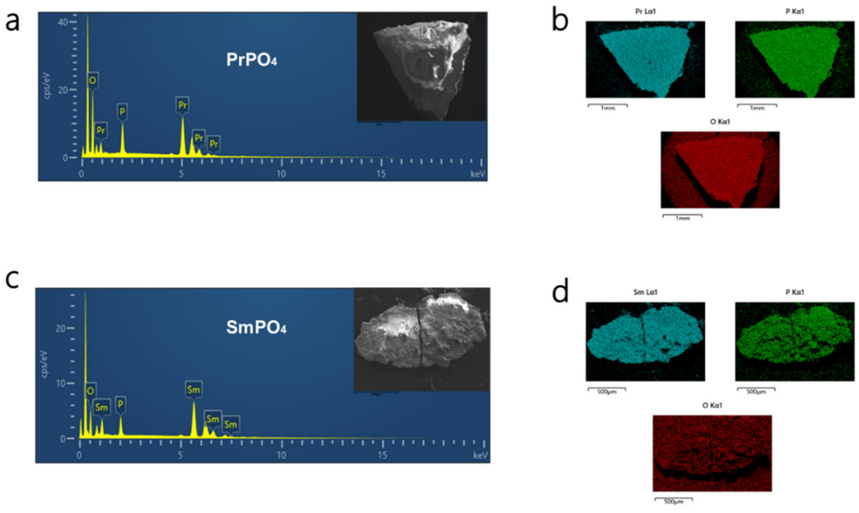

Supplementary Figure S4. Element distribution of the surfaces in PSC $PrPO_4$ and $SmPO_4$ monoliths. a−b, The EDS of $PrPO_4$ and the corresponding mapping images: Pr Lα1, P Kα1 and O Kα1. c−d, The EDS of $SmPO_4$ and the corresponding mapping images: Sm Lα1, P Kα1 and O Kα1.

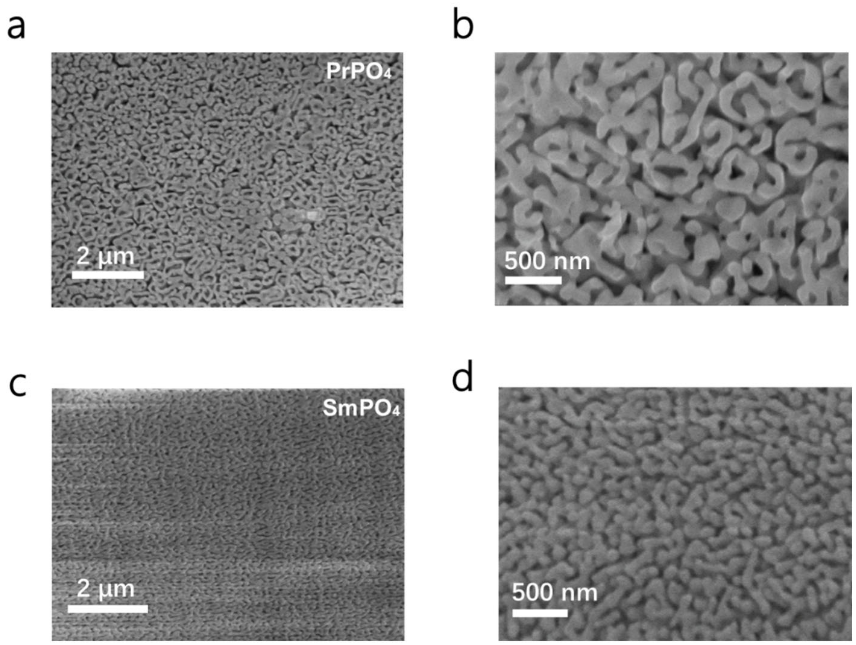

Supplementary Figure S5. Microstructure of the PSC $PrPO_4$ and $SmPO_4$ monoliths. a−b, The SEM of porous $PrPO_4$ single crystal. c−d, The SEM of porous $SmPO_4$ single crystal.

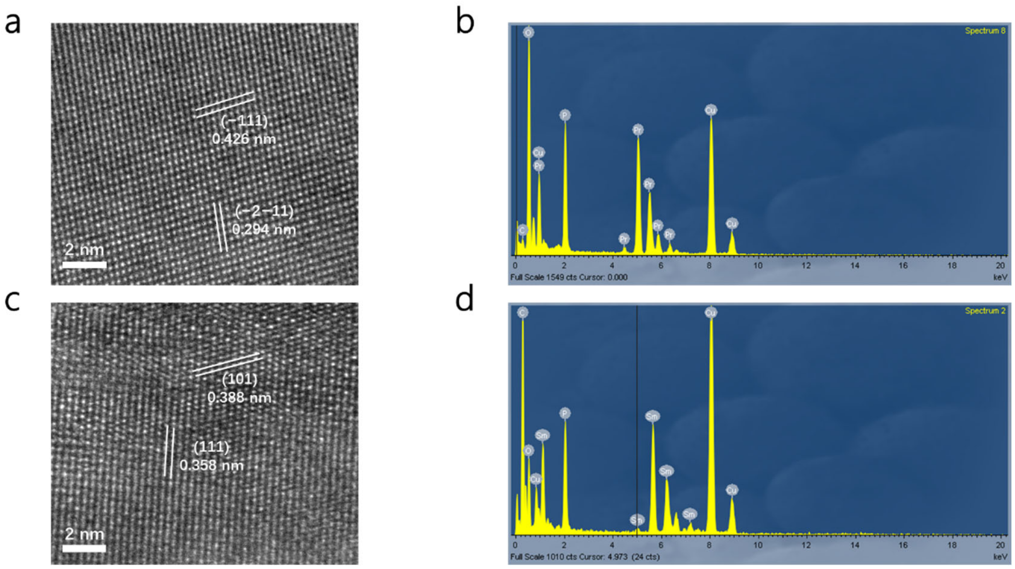

Supplementary Figure S6. Microstructure and element distribution of PSC $PrPO_4$ and $SmPO_4$. a−b, Enlarged HRTEM and the corresponding EDS of PSC $PrPO_4$. c−d, Enlarged HRTEM and the corresponding EDS of PSC $SmPO_4$.

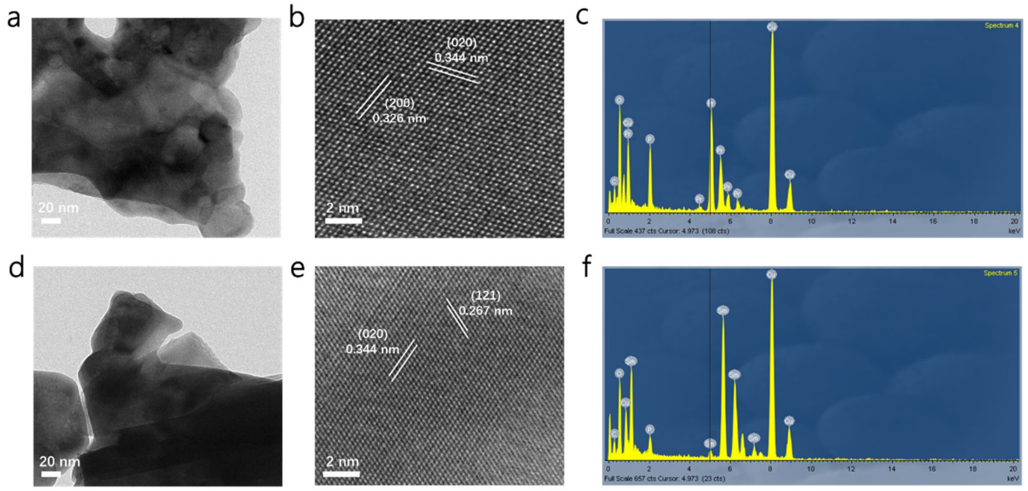

Supplementary Figure S7. Microstructure and element distribution of PSC $PrPO_{4-x}$ and $SmPO_{4-x}$. a−c, TEM, enlarged HRTEM and the corresponding EDS of PSC $PrPO_{4-x}$. d−f, TEM, enlarged HRTEM and the corresponding EDS of PSC $SmPO_{4-x}$.

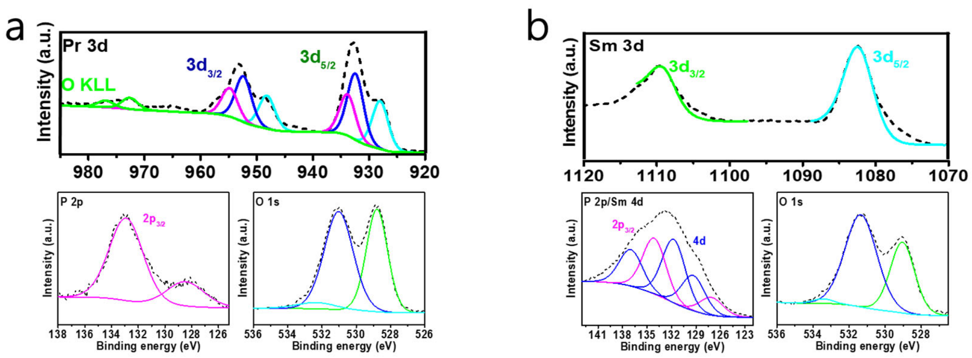

Supplementary Figure S8. Chemical state of the surfaces in PSC $PrPO_{4-x}$ (a) and PSC $SmPO_{4-x}$ (b).

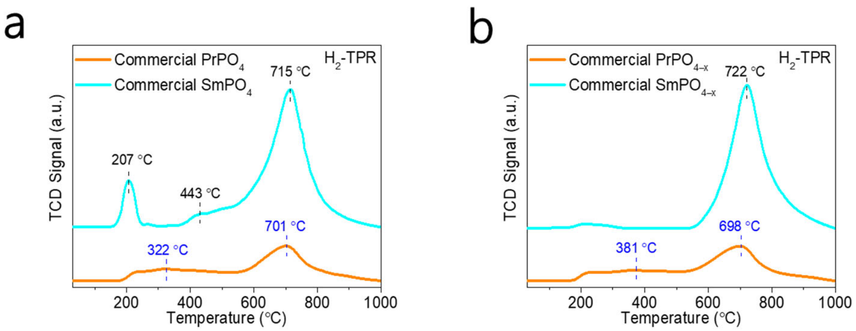

Supplementary Figure S9. Reduction characterization of commercial $PrPO_4$, $SmPO_4$, $PrPO_{4-x}$ and $SmPO_{4-x}$. a, H_2-TPR for commercial $PrPO_4$ and $SmPO_4$. b, H_2-TPR for commercial $PrPO_{4-x}$ and $SmPO_{4-x}$

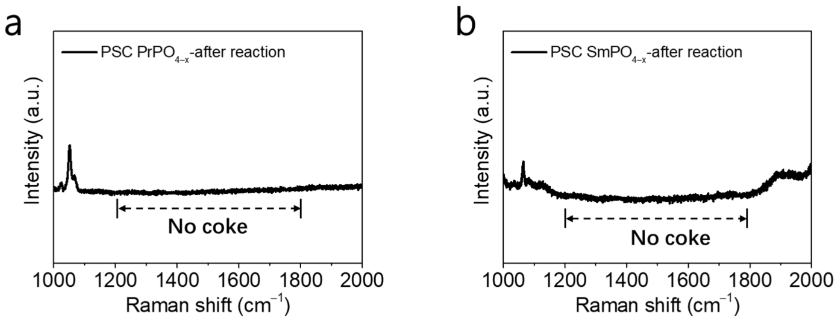

Supplementary Figure S10. Characterization of PSC $PrPO_{4-x}$ and PSC $SmPO_{4-x}$ after reaction. a−b, Raman spectra.

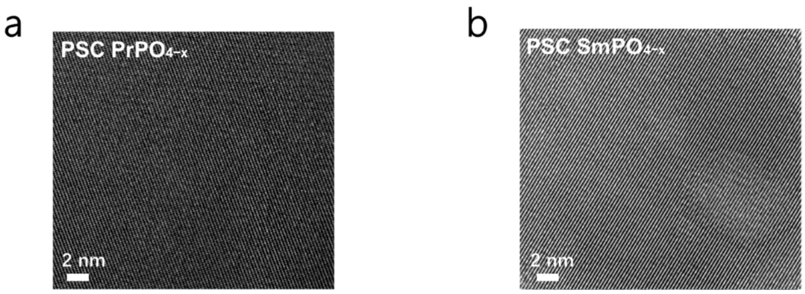

Supplementary Figure S11. Microstructure of PSC $PrPO_{4-x}$ and PSC $SmPO_{4-x}$ after long-term reaction. a−b, TEM images.

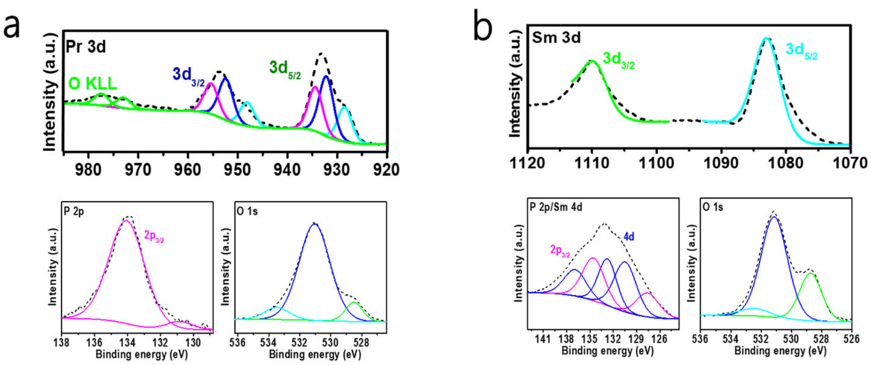

Supplementary Figure S12. Chemical state of the surfaces in PSC $PrPO_{4-x}$ (a) and PSC $SmPO_{4-x}$ (b) after long-term reaction.

Table S1. Porosity level analysis from Mercury intrusion method of commercial $PrPO_4$, commercial $SmPO_4$, PSC $PrPO_4$ and PSC $SmPO_4$.

Sample	Assembly Mass (g)	Penetrometer Mass (g)	Sample Mass (g)	Mercury Volume (mL)	Penetrometer Volume (mL)	Sample Volume (mL)	Total Intrusion Volume (mL/g)	Sample Actual Pressed Mercury Volume (mL)	Porosity (%)
Commercial $PrPO_4$	137.5470	62.4647	0.6518	5.4997	6.0536	0.5539	0.6039	0.3954	71.3847
PSC $PrPO_4$	135.0230	63.2316	0.3906	5.2759	5.8745	0.5986	1.3411	0.5238	87.5042
Commercial $SmPO_4$	141.3836	62.5565	0.3315	5.8001	6.0536	0.2535	0.4638	0.1537	60.6312
PSC $SmPO_4$	138.5242	62.4689	0.3645	5.5928	6.0536	0.4608	1.1018	0.4016	87.1528
